# Preclinical dose response study shows *NR2E3* can attenuate retinal degeneration in the retinitis pigmentosa mouse model *Rho*^*P23H+/*^^−^

**DOI:** 10.1038/s41434-024-00440-6

**Published:** 2024-01-26

**Authors:** Shannon M. McNamee, Natalie P. Chan, Monica Akula, Marielle O. Avola, Maiya Whalen, Kaden Nystuen, Pushpendra Singh, Arun K. Upadhyay, Margaret M. DeAngelis, Neena B. Haider

**Affiliations:** 1 Schepens Eye Research Institute, Massachusetts Eye and Ear, Department of Ophthalmology, Harvard Medical School, Boston, MA USA; 2 University of Massachusetts Amherst, Amherst, MA USA; 3 Ocugen, Inc, Malvern, PA USA; 4 Department of Ophthalmology, Jacobs School of Medicine & Biomedical Sciences, University at Buffalo, Buffalo, NY USA

**Keywords:** Gene expression, Cell biology

## Abstract

Retinitis pigmentosa (RP) is a heterogeneous disease and the main cause of vision loss within the group of inherited retinal diseases (IRDs). IRDs are a group of rare disorders caused by mutations in one or more of over 280 genes which ultimately result in blindness. Modifier genes play a key role in modulating disease phenotypes, and mutations in them can affect disease outcomes, rate of progression, and severity. Our previous studies have demonstrated that the nuclear hormone receptor 2 family e, member 3 (*Nr2e3*) gene reduced disease progression and loss of photoreceptor cell layers in *Rho*^*P23H*^^−^^*/*^^−^ mice. This follow up, pharmacology study evaluates a longitudinal *NR2E3* dose response in the clinically relevant heterozygous *Rho*^*P23H*^ mouse. Reduced retinal degeneration and improved retinal morphology was observed 6 months following treatment evaluating three different *NR2E3* doses. Histological and immunohistochemical analysis revealed regions of photoreceptor rescue in the treated retinas of *Rho*^*P23H+/*^^−^ mice. Functional assessment by electroretinogram (ERG) showed attenuated photoreceptor degeneration with all doses. This study demonstrates the effectiveness of different doses of *NR2E3* at reducing retinal degeneration and informs dose selection for clinical trials of *Rho*^*P23H*^-associated RP.

## Introduction

Retinitis pigmentosa (RP) is the most common form of inherited blindness. RP is a heterogeneous disease that varies in age of onset, rate of progression and genetic etiology based on the mutation and gene impacted [1–5]. Retinitis pigmentosa affects 1 in 4000 individuals worldwide [6, 7]. RP diseases include syndromic and non-syndromic forms with over 200 unique genetic mutations associated with disease onset [8–14]. There is currently only one type of treatment approved to treat a specific form of RP, voretigene neparvovec (Luxturna^®^) is approved for treatment in patients with biallelic *RPE65* gene mutations [15–18]. Additionally, over 40% of RP cases cannot be genetically diagnosed [19]. While there is great heterogeneity in RP disease, the common shared pathology is degeneration of photoreceptor (PR) cells. The genes and mechanisms causing photoreceptor degeneration can vary and the cumulative degenerative outcome is influenced by a mutational load on the system that includes the primary mutation and modifier genes among other factors [20]. Our previous publication revealed the novel finding that the modifier gene *Nr2e3* can treat retinal degeneration in several mouse models of RP [19].

Modifier genes are defined as allelic variants within a normal population, and can significantly impact the onset, progression, and severity of diseases [21–24]. Studies of multiple diseases including spinal muscular atrophy, spinocerebellar ataxia type 1, dystonia, epileptic encephalopathy, cystic fibrosis, and retinal degeneration show the effect of modifier genes and their impact on disease phenotypes altered by shifted genetic backgrounds [25–33]. Mutations in the modifier gene nuclear hormone receptor *NR2E3* are associated with several types of retinal degeneration including clumped pigmentary retinal degeneration (CPRD), Goldmann–Favre syndrome (GFS), enhanced S-cone syndrome (ESCS), and autosomal dominant retinitis pigmentosa (adRP) [34]. The variable phenotypes of *NR2E3*-associated retinal degeneration suggest modifier genes could be influencing disease manifestation and outcomes. *Nr2e3* plays a major role in the retina by regulating the development and maintenance of photoreceptor cells, and regulating gene networks in pathways including ER stress, neuroprotection, photoreceptor function, apoptosis, immune response, and cell survival, and thereby impacting homeostasis of the retina [19, 35–41]. Modifier genes such as *NR2E3* could thus be a more effective therapeutic agent and especially beneficial in RP cases where the primary mutation cannot be identified. Previous publications by our lab examined the effectiveness of *Nr2e3* as a therapeutic for *NR2E3*-associated RP as well as other forms of RP that do not possess an *NR2E3* mutation [19].

Although RP is a rare disease, mutations in the Rhodopsin gene represent about 40% of all adRP cases [42]. Additionally, there are more than 150 known mutations in Rhodopsin and the *Rho*^*P23H*^ mutation accounts for about 10% of *Rho* related adRP in the United States alone [42–45]. The *Rho*^*P23H*^ mutation creates a misfolded protein that is not processed properly and accumulates in the endoplasmic reticulum (ER) [19, 45]. The accumulation of these aberrant *Rho* proteins causes cellular stress [43, 46–48]. Patients with this mutation usually experience night-blindness followed by a progressive loss of their visual field [42–48]. This was a pharmacological study to demonstrate the efficacy of dosage and longitudinal impact of *NR2E3* modifier gene therapy to treat retinal degeneration in the *Rho*^*P23H+/*^^−^ mouse when administered during early/intermediate degeneration. Treated animals were assessed 1, 3, and 6 months after dose administration to track the degree of longitudinal rescue of degeneration. The *Rho*^*P23H*^ mouse is a model for human Retinitis Pigmentosa 4 (RP-4). These mice harbor a substitution of the amino acid proline with histidine at position 23 resulting in protein misfolding and degradation from an aberrant message [19, 45]. Heterozygous *Rho*^*P23H*^ mice were used for this study, as they are more clinically relevant with adRP features including gradual loss of rods and scotopic ERG function followed by loss of cones and photopic ERG function [45, 49, 50]. *Rho*^*P23H+/*^^−^ mice exhibit rapid photoreceptor degeneration from postnatal day (P) 15 to P30 that progresses to gradual degeneration over time [45, 49, 50]. The outer nuclear layer (ONL) thickness of a P30 *Rho*^*P23H+/*^^−^ retina is about 30–40% less than a normal wild-type retina and continues to gradually decrease with age [45, 49]. *Rho*^*P23H+/*^^−^ mice retain a majority of their photoreceptors; however, the cells in the inner and outer segments show structural disorganization and as mice age the outer segments (OS) become shorter [44]. P35 mice possess shorter rod OS and by P63, have about half the normal amount of rod nuclei compared to wild-type mice [45]. Scotopic ERG responses are severely reduced by P41 and are nearly undetectable by P170 [45]. In comparison, homozygous mice exhibit severe rapid retinal degeneration by P23 with almost complete loss of photoreceptors by P63 [45]. It is important to note, our previous study of *Nr2e3* therapeutic utilized *Rho*^*P23H*^^−^^*/*^^−^ mice which possess an abnormal fundus phenotype whereas this study utilized *Rho*^*P23H+/*^^−^ mice that have a normal fundus phenotype [19]. This study demonstrates that low, mid, and high doses of AAV5-*hNR2E3* can slow retinal degeneration in the *Rho*^*P23H+/*^^−^ mouse for at least 6 months following treatment when administered during early/intermediate disease.

## Materials and methods

### Animal maintenance

This study was performed in accordance with the Association for Research in Vision and Ophthalmology (ARVO) Statement for the Use of Animals in Ophthalmic and Vision Research and the Guide for the Care and Use of Laboratory Animals of the National Institute of Health. The Schepens Eye Research Institute vivarium housed and bred animals under standard conditions, 68-74 °F and 12-h light/dark cycle, for the course of this study. All animals and procedures used in this study were approved by the Schepens Eye Research Institute Animal Care and Use Committee (Protocol Number: 2020N000178) in accordance with the Animal Welfare Act Regulations. B6.129S6(Cg)-Rhotm1.1Kpal/J (*Rho*^*P23H*^^−^^*/*^^−^; Jax stock #017628) [45] and C57BL6/J (B6, Jax stock #000664) mice were obtained from Jackson Laboratories, Bar Harbor, ME. *Rho*^*P23H +/*^^−^ animals were generated by crossing *Rho*^*P23H*^^−^^*/*^^−^ knock-in males with B6 females [51].

### Scientific rigor and reproducibility

Estimation of the required sample size for each analysis and quantification was conducted via a power calculation using G*Power 3.1 software analysis. 90% power and 30% difference with a significance level of 0.05 was provided by a minimum of eight animals per experimental group according to means and standard deviations defined in previous publications. All procedures performed in this study used a standardized protocol for consistent replication. Bias was avoided using a randomized and double blinded study method with several trained individuals. A minimum of 8 biological replicates were performed per dose and timepoint for statistical significance. Exclusion criteria included animals with unresolved surgical trauma, premature unintended death, or cataracts. Approximately 10% of the 100 animals used in this study were excluded from the results. No gender bias was observed between males (~48.04%) and females (~51.96%) used in this study.

### Statistical analysis

Mean ± standard error of the mean (SEM) represents technical variations in rescue in electroretinography and outer nuclear layer thickness due to focal delivery of *NR2E3*. Statistical significance was analyzed in graphs using either two-way analysis of variance (ANOVA) or *t* test. Cell count analysis was performed to analyze the difference between untreated and each dose treated retinas 6 months after treatment. The mean peak scotopic A- and B-wave amplitudes of untreated and treated animals for each dose and timepoint were compared.

### Genotyping

The quick lysis sodium hydroxide method was used for DNA isolation of tail biopsies from the *Rho*^*P23H+/*^^−^ mice. Samples were amplified with the transgene specific primers P23H-F: GGTAGCACTGTTGGGCATCT and P23H-R: GACCCCACAGAGACAAGCTC [52]. Approximately 50 ng of DNA was used for polymerase chain reaction (PCR) amplification in a reaction volume of 10 μL. Reactions consisted of 10 μM each of forward and reverse primer, 40 mM of dNTP mix, 10x buffer with MgCL_2_, and 5 U/ml AmpliTaq DNA polymerase. Reaction parameters were 94 °C for 2 min followed by 39 cycles at 94 °C for 15 s, 60 °C for 30 s, and 72 °C for 1 min ending with 72 °C for 5 min. 2% ethidium bromide-stained agarose gels were used to separate amplicons and visualize bands under UV light. Wild-type amplicon is 150 base pairs (bp), and the mutant amplicon is 320 bp. The large difference in amplicon size is due to the amplification of the knock-in transgene in mutant mice [52]. Heterozygous animals were genotyped using the protocol recommended by Jackson Laboratory, Bar Harbor, ME.

### AAV5-*hNR2E3* dose preparation

Three doses were evaluated. The low dose was chosen to be one log unit lower than the therapeutic (mid) dose to examine the lower limit of dose effectiveness, while the high dose was chosen to be 4 times greater than the mid to evaluate potential dose toxicity. The human *NR2E3* gene was packaged into AAV5 capsid with optimized regulatory elements to create the drug product (AAV5-*hNR2E3*). Ocugen, INC. supplied the GLP vector drug product and formulation buffer (10 mM sodium phosphate, 180 mM sodium chloride, and 0.001% Poloxamer 188 (PF-68) at pH 7.3 ± 0.5). The drug product was diluted with formulation buffer to achieve the following concentrations: 2 × 10^11^ Vg/ml, 2 × 10^12^ Vg/ml, 8 × 10^12^ Vg/ml. The intended low (1 × 10^8^ vgc/eye), mid (1 × 10^9^ vgc/eye), and high (4 × 10^9^ vgc/eye) doses used for subretinal injection were aliquoted from these dilutions.

### Subretinal injection

All AAV5-*hNR2E3* doses were delivered via subretinal injection into the right eye of *Rho*^*P23H+/*^^−^ mice. P30 mice were anesthetized with ketamine/xylazine by intraperitoneal (IP) injection using a 0.5” 25G needle. An incision was made in the sclera with a 25G needle and a total volume of 0.5 µL of either low, mid, or high dose AAV5-*hNR2E3* was manually administered into the subretinal space [19]. The contralateral (left) eye of treated animals was used as the untreated control in this study. The untreated eye received either no injection, a mock injection with no vector, or a mock injection with saline buffer to demonstrate that the injection itself does not impact the results.

### Clinical examination

Fundus imaging of the clinical phenotype was performed using a Micron IV Retinal Imaging Camera and Stream Pix software (Phoenix Research Laboratories, Pleasanton, CA, USA) on treated and untreated *Rho*^*P23H+/*^^−^ mice. After administering anesthesia via IP injection of ketamine/xylazine with a 25G needle, 1 drop of 1% Tropicamide was applied to dilate pupils and Genteal was applied to moisturize the eye. Mice were then placed on a platform and aligned using a real-time fundus image of the retina to center the optic nerve before capturing [15].

### Electroretinography

Electroretinography (ERG) analysis was performed on AAV5-*hNR2E3* treated and untreated mouse eyes with the Espion Visual Electrophysiology System (Diagnosys LLC, Lowell, MA, USA). Mice were dark-adapted overnight in a dark chamber and anesthetized with ketamine/xylazine by IP injection using a 0.5” 25G needle. Pupils were dilated with a drop of 1% Tropicamide and kept moist using a topical application of Genteal. Mice were positioned on to the platform and the ground electrode was placed at the base of the tail followed by the reference electrode placed just underneath the skin of the scalp. Two gold loop electrodes (Diagnosys LLC) were aligned and placed flush on the eyes of the mouse. Dark- and light-adapted ERGs were performed and recorded using the previously described protocol [19]. Peak scotopic A- and B-wave amplitudes were taken from each animal and averaged for each dose and timepoint compared to the untreated and wild-type amplitudes.

### Histology

Hematoxylin and eosin (H/E) staining of retinal layers was performed using a standard protocol established in the lab [19]. Following euthanasia, eyes were immediately cauterized to mark dorsal/ventral orientation and to allow penetration of fixative. Eyes were fixed in 4% paraformaldehyde (PFA) in 1x PBS overnight at 4 °C and paraffin embedded. 5 µm serial sections were cut across 100 µm of retinal depth. Sections were deparaffinated in xylene and ethanol dips. Then, sections were stained with Harris hematoxylin and eosin Y. Coverslips were mounted with Permount and images were taken using a Leica DMI6000 microscope (Leica Microsystems, Wetzlar, Germany). A minimum of 3 personnel counted photoreceptor cell layers in the outer nuclear layer (ONL) in a double blinded method [19, 53]. Photoreceptor rescue was quantified by comparing the number of cell layers in the ONL of *NR2E3* treated and untreated *Rho*^*P23H+/*^^−^ retinas and B6 control retinas.

### Immunohistochemistry

Immunohistochemical (IHC) analysis was performed on 5 µm paraffin embedded sections using standardized lab protocols [19]. Sections were deparaffinated in a series of xylene and ethanol dips followed by a rinse in PBS. Antigen sites were unmasked by soaking the sections in sodium citrate buffer. Sections were blocked with 2% normal horse serum (S-2000 Vector Labs, CA) in 1× PBS at room temperature for an hour. The primary antibodies rhodopsin (mouse monoclonal, Millipore MAB5356), blue opsin (rabbit polyclonal, Millipore AB5407), and green/red opsin (rabbit polyclonal, Millipore AB5405) were applied at 1:200 dilutions in PBS and incubated at 4 °C overnight. The following day sections were rinsed with PBS and the corresponding secondary antibody was applied in the dark in a 1:400 dilution in PBS: Alexa Fluor 488 goat anti-mouse (Invitrogen A11001) for rhodopsin and Alexa Fluor 488 goat anti-rabbit (Invitrogen A11008) for green and blue opsin slides. These slides were then incubated for an hour in the dark and finally nuclei were labeled with 4,6-Diamidino-2-Phenylindole, Dihydrochloride (DAPI, dilactate, Invitrogen D3571). Opsin labeling was visualized and imaged using a Leica DMI6000 fluorescent microscope (Leica Microsystems, Wetzlar, Germany) [19].

## Results

### AAV5-*hNR2E3* delivery in three different doses in *Rho*^*P23H+/*^^−^ animals rescues photoreceptor degeneration for at least 6 months

**Fig. 1:**
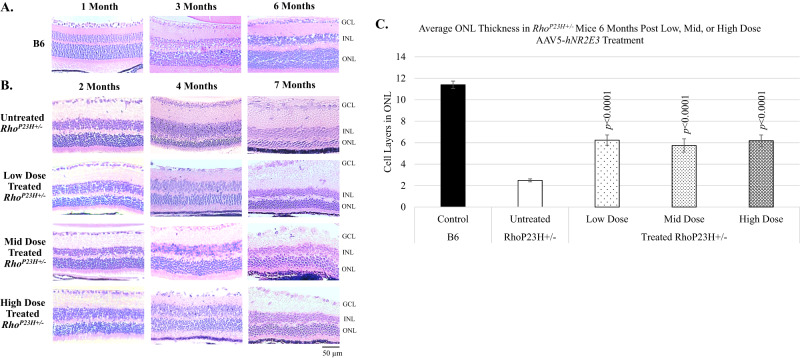
Treatment with AAV5-*hNR2E3* slows ONL degeneration in *Rho*^*P23H+/*^^−^ retinas for all doses. ONL thickness in untreated *Rho*^*P23H+/*^^−^ animals compared to the thickness of rescued regions in AAV5-*hNR2E3* treated *Rho*^*P23H+/−*^ mice. **A** C57BL/6J (B6) normal histology of 1-, 3-, and 6-month retinas. **B** Comparison of untreated and *NR2E3* treated *Rho*^*P23H*+/^^−^ animals injected at 1 month of age and collected at 1-, 3-, and 6-months post-injection (animal ages 2, 4, and 7 months). **C** Wild type and untreated *Rho*^*P23H+/*^^−^ retinas (7 months old) counted in comparison to 6-months post-treatment (animal ages 7 months) counts of 1-month *NR2E3* treated *Rho*^*P23H+/*^^−^ retinas. *p* < 0.0001 for all 3 doses compared with untreated *Rho*^*P23H+/*^^−^ eyes. Serial sections of central retina counted over 100 µm. Results are mean ± SEM. Low Dose 1 × 10^8^ vg/eye, Mid Dose 1 × 10^9^ vg/eye, High Dose 4 × 10^9^ vg/eye. GCL: Ganglion Cell Layer; INL: Inner Nuclear Layer; ONL: Outer Nuclear Layer. Scale bar = 50 µm. *N* ≥ 4.

*NR2E3* was administered in a low, mid, or high dose by subretinal delivery during early/intermediate disease progression and photoreceptor rescue was evaluated 1-, 3-, and 6-months post-treatment in *Rho*^*P23H+/*^^−^ mice. The retina of a normal mouse is composed of the ONL (10–12 layers of rod and cone photoreceptor nuclei), inner nuclear layer (INL, 5–6 layers of inner retinal cells), and a single ganglion cell layer (GCL) [19]. *Rho*^*P23H+/*^^−^ mice lose 30–40% of ONL thickness by P30 followed by gradual decreases until about 6 months of age [45, 49]. Histological analysis of *Rho*^*P23H+/*^^−^ animals dosed with AAV5-*hNR2E3* at P30 showed improved photoreceptor survival up to 6 months after treatment. H/E staining showed improved ONL thickness in treated retinas compared to untreated retinas for the 3- and 6-months post timepoints (Fig. 1B). The ONL thickness was sustained across the disease progression from 2 months (1-month post-treatment) to 7 months (6-months post-treatment) of age. Results indicate that all three *NR2E3* doses can reduce degeneration and sustain photoreceptor survival in *Rho*^*P23H+/*^^−^ mice for at least 6 months after treatment. Counts of the photoreceptor nuclei layers in the ONL of *Rho*^*P23H+/*^^−^ animals treated at P30 and collected 6 months later showed that photoreceptor survival was significantly increased compared to untreated animals (Fig. 1C; *p* < 0.0001). All three doses showed rescue with a p-value less than 0.0001. The ONL thickness did not return to the normal range indicating that degeneration is likely not being reversed but that treatment with *NR2E3* during early/intermediate disease can slow or halt degeneration.

### *NR2E3* dose therapy preserved cone and rod opsin expression in *Rho*^*P23H+/*^^−^ animals up to 6 months after treatment during early/intermediate disease progression

**Fig. 2:**
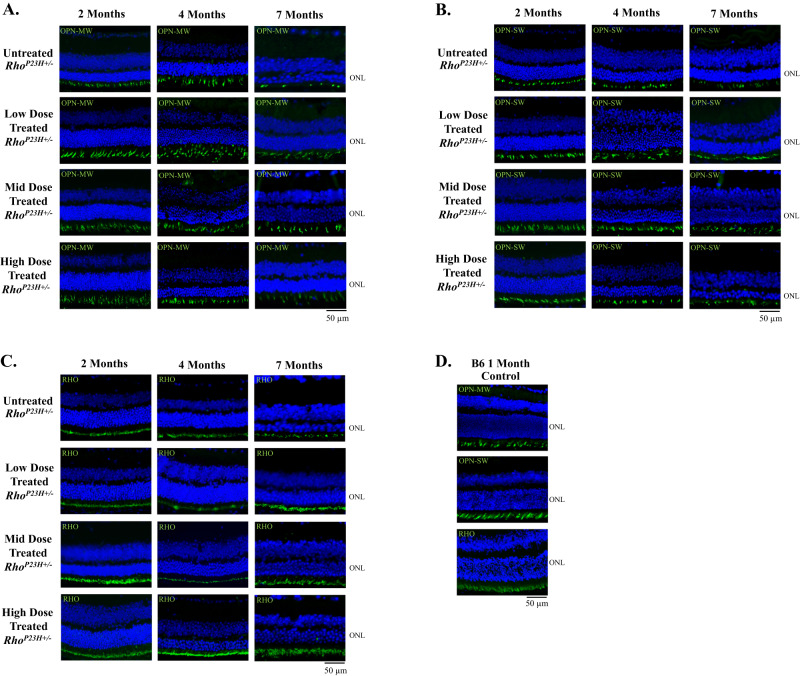
Treatment with low, mid, or high dose AAV5-*hNR2E3* maintained opsin expression in *Rho*^*P23H+/*^^−^ animals. Expression comparison between untreated and *NR2E3* treated *Rho*^*P23H+/*^^−^ animals injected at 1 month of age and evaluated 1-, 3-, and 6-months post-injection labeled with **A** green opsin (OPN-MW), **B** blue opsin (OPN-SW), and **C** rhodopsin (RHO) antibodies. **D** C57BL/6J (B6) normal expression of green opsin (OPN-MW), blue opsin (OPN-SW), and rhodopsin (RHO). Green opsin (OPN-MW) and blue opsin (OPN-SW) labeling showed maintained expression in treated animals. Rhodopsin (RHO) labeling showed restored expression in treated animals. Dapi is indicated in blue and opsins are shown in green. Low Dose 1 × 10^8^ vg/eye, Mid Dose 1 × 10^9^ vg/eye, High Dose 4 × 10^9^ vg/eye. Scale bar = 50 μm. *N* = 8.

Immunohistochemical analysis examined the longitudinal efficacy of a low, mid, and high dose of AAV5-*hNR2E3* to rescue cone opsin and rhodopsin expression in *Rho*^*P23H+/*^^−^ mice. In the *Rho*^*P23H+/*^^−^ mouse, about half of rod photoreceptor nuclei are lost by 2 months of age followed by slow progressive loss of cones until around 6 months of age [45]. *Rho*^*P23H+/*^^−^ animals were dosed at P30 with *NR2E3*, and eyes were collected at 1-, 3-, and 6- months post-treatment. Treated animals showed preserved expression of all opsins compared to untreated animals for all doses lasting up to 6 months after treatment (Fig. 2). Most notable was the preservation of rhodopsin expression in *NR2E3* treated eyes compared to untreated. This can be attributed to the age of *NR2E3* administration. Since almost half of all rods are lost by 2 months of age, administration of the therapeutic at P30 can rescue a majority of the rod photoreceptors from degeneration. Expression of all opsins was observed in all timepoints and doses for treated *Rho*^*P23H+/*^^−^ mice compared to untreated but was not restored to normal levels 6 months later.

### Three different doses of AAV5-*hNR2E3* preserved photoreceptor function in *Rho*^*P23H+/*^^−^ animals treated during early/intermediate disease

**Fig. 3:**
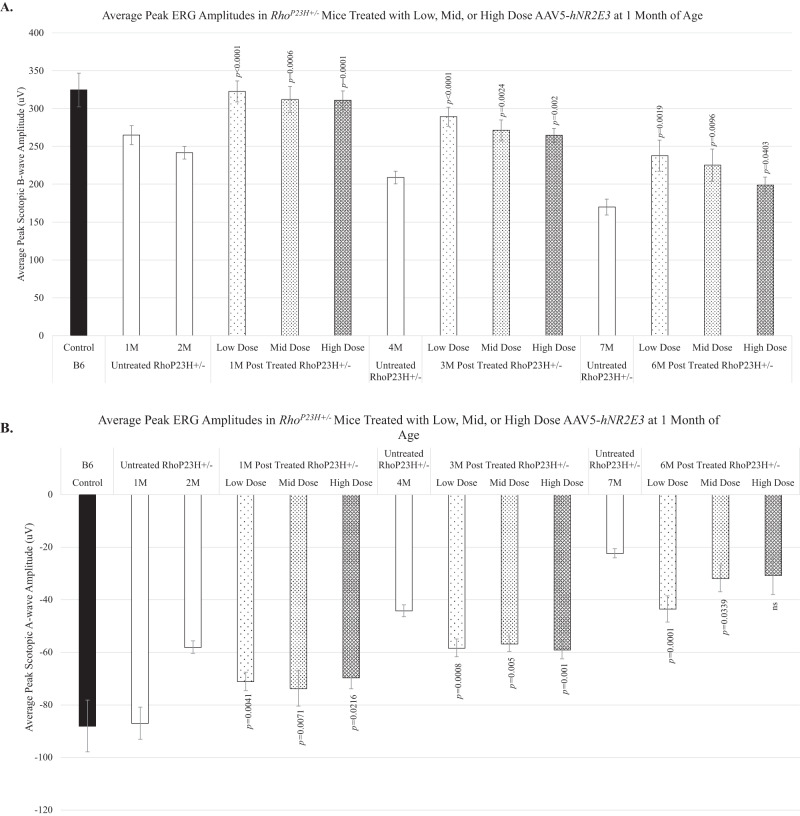
AAV5-*hNR2E3* treated *Rho*^*P23H+/*^^−^ mice showed preserved retinal function for all doses. *Rho*^*P23H+/*^^−^ mice were treated with low, mid, or high dose *NR2E3* at 1 month of age and assessed 1-, 3-, and 6-months post-treatment (2, 4, and 7 months of age) with ERG. **A** There was a significant increase in peak scotopic B-wave amplitude of treated vs untreated animals at 1 month (*p* < 0.0006–0.0001), 3 months (*p* < 0.002–0.0001) and 6 months (*p* < 0.05–0.002) post-treatment. **B** Scotopic A-wave peak amplitudes were significantly higher in treated *Rho*^*P23H+/*^^−^ mice compared to untreated 1 month (*p* ≤ 0.0216–0.0041), 3 months (p ≤ 0.005–0.0008), and 6 months (*p* ≤ 0.0339–0.0001) post-treatment. Low Dose 1 × 10^8^ vg/eye; Mid Dose 1 × 10^9^ vg/eye, High Dose 4 × 10^9^ vg/eye. Results are mean ± SEM. *N* ≥ 5.

Dark-adapted ERG analysis was performed to examine the efficacy of low, mid, and high doses of AAV5-*hNR2E3* at rescuing rod- and cone-photoreceptor driven responses. Disease progression in the *Rho*^*P23H+/*^^−^ mouse model results in the progressive loss of photoreceptor function with severely diminished scotopic ERG responses by around 6 months of age [45]. Through our previous studies, we were able to show improved ERG responses in *Rho*^*P23H*^^−^^*/*^^−^ mice treated with *Nr2e3* [19]. A- and B-wave peak amplitudes were analyzed to evaluate photoreceptor functionality for perceiving light and transmitting the signal. The A-wave response measures the ability of photoreceptors to receive light and transform that information into an electrical signal. B-wave responses measure the ability of photoreceptors to send the electrical signal to second-order neurons [54]. *Rho*^*P23H+/*^^−^ animals treated at P30 maintained normal levels of scotopic peak B-wave amplitudes in treated retinas compared to untreated retinas lasting over 6 months after treatment (Fig. 3A; *p* < 0.0403–0.0001). The most robust amplitudes were apparent 1-month post-treatment for all doses with the low (Fig. 3A; *p* < 0.0001) and high (Fig. 3A; *p* = 0.0001) doses having the most significant preservation of B-wave at that time. The low dose continued showing the most significant B-wave preservation of the three doses at the 3- and 6-month post-treatment timepoints. Noteworthy, ERG responses decreased over time for all doses of *NR2E3* treatment but remained significant and within normal levels for at least 6 months after treatment compared to untreated *Rho*^*P23H+/*^^−^. This suggests a significant attenuation of degeneration as the treated values still remain above or within the values of the 2-month untreated responses with the exception of the 6-months post high dose. Similar to the B-wave trends, the 1-month post timepoint showed the most robust preservation of peak scotopic A-wave amplitudes and the low dose showed the most significant preservation of amplitudes at all timepoints (Fig. 3B; *p* ≤ 0.0041–0.0001). Similarly to the B-waves, the A-wave amplitude preservation decreased over time for all doses supporting the notion that degeneration is slowed significantly by *NR2E3* treatment.

## Discussion

Diseases such as RP present a major obstacle to the medical community. The large phenotypic variations in RP stemming from factors like environment, epigenetics, gene mutations, and allelic heterogeneity make finding effective treatments difficult [55]. However, there is great promise in modifier genes as therapeutics for various forms of RP. The modifier gene *Nr2e3*, which has a key role in several homeostasis pathways such as phototransduction, cell survival, ER stress, oxidative stress, apoptosis, immunity, metabolism, and neuroprotection, has been used in previous studies to reduce disease progression and even rescue retinal degeneration [19, 56]. Our previous publication revealed that *Nr2e3* gene therapy can rescue degeneration in several models of RP including the more severe *Rho*^*P23H*^^−^^*/*^^−^ mouse. Adeno-associated virus serotype 5 (AAV5) was the chosen viral vector for this study based on our previous publication that tested gene delivery with AAV2.7m8, AAV5 and AAV8 as well as its safety and efficiency at transducing retinal pigment epithelium (RPE) and photoreceptor cells [19, 57–63]. This was a pharmacological study to inform the longitudinal effectiveness of several doses of AAV5-*hNR2E3* in *Rho*^*P23H+/*^^−^ mice for treating *Rho*^*P23H*^-associated RP. The novel components of this study were the examination of a low, mid, and high dose of AAV5-*hNR2E3*, examination of dose effectiveness up to 6 months after administration, and the use of the heterozygous *Rho*^*P23H*^ mouse model instead of the more severe homozygous model from our previous study. The heterozygous model is more representative of both the genotype and phenotype of human *Rho*^*P23H*^-associated RP. Analysis showed significant improvement of photoreceptor survival and function in *Rho*^*P23H+/*^^−^ mice for all three doses lasting at least 6 months after administration of *NR2E3* therapeutic. Preserved expression of blue opsin, green opsin, and rhodopsin proteins was also observed in all doses for up to 6 months. Note that H/E and immunohistochemistry revealed regions of rescue in the treated retinas due to the focal delivery of *NR2E3* into the subretinal space. Based on this evidence, histological, molecular, and functional analysis showed that *NR2E3* overexpression can slow degeneration and improve the disease outcome over time in the *Rho*^*P23H+/*^^−^ mouse. Clinical examination of the fundus of treated vs. untreated *Rho*^*P23H+/*^^−^ mice 1-, 3-, and 6-months post-treatment revealed no adverse effects on the retina as a result of *NR2E3* therapy (Supplemental Fig. 1). Additionally, no abnormal systemic responses or overt toxic effects were observed in daily behavior or gross pathology following therapy administration. Comparison of untreated control *Rho*^*P23H+/*^^−^ fundus and H/E data demonstrated no obvious impacts of the injection itself on the retina in eyes that received either a mock injection, mock and saline buffer injection, or no injection (Supplemental Fig. 2).

*Rho*^*P23H+/*^^−^ mice are functional nulls in which the protein does not undergo complete glycosylation, becomes trapped, and degrades within the endoplasmic reticulum (ER) and/or Golgi apparatus [19, 45]. *Nr2e3* has been shown in recent studies to play a role in the ER stress pathway. More specifically it is believed to target inositol-requiring enzyme 1 (*Ire1*), a cell survival and ER stress factor, which was found in our previous publication to be upregulated following *Nr2e3* administration in various models of RP including *Rho*^*P23H*^^−^^*/*^^−^ [19]. ER stress activates *Ire1* which encodes for an ER-resident transmembrane protein and is a key regulator of the most highly conserved branch of the unfolded protein response (UPR) signaling pathway [64–66]. *Ire1* activation helps to clear misfolded proteins from the ER including misfolded *Rho*^*P23H*^ by upregulating several ER chaperones that partially ameliorate the misfolding defect in *Rho*^*P23H*^ proteins [46–48, 66]. Therefore, upregulation of *Ire1* via *NR2E3* administration may prevent the degradation of *Rho*^*P23H*^ protein and promote escape from the ER into the retinal outer segments thus delaying degeneration from ER stress.

Unlike our prior study on *Nr2e3* treatment in *Rho*^*P23H*^^−^^*/*^^−^ mice at P0 (prior to disease onset) and P21 (early/intermediate disease progression), this study examined administration of *NR2E3* therapy at P30, which falls in early/intermediate disease, in heterozygous *Rho*^*P23H*^ mice. Treatment prior to disease onset or as early into disease progression as possible is obviously the optimal treatment time for the majority of therapeutics and diseases as the amount of damage done by disease is minimal. However, this is often not feasible in the clinical setting as diseases are not usually diagnosed until symptoms arise, which can be in the early to intermediate stages of disease when significant damage has already occurred in systems. The results of this study demonstrate that *NR2E3*, while more robust when administered prior to onset, can reduce degeneration in the early/intermediate stages of disease and for at least 6 months after treatment. It is also important to note that *NR2E3* maintained ERG responses in *Rho*^*P23H+/*^^−^ animals at normal levels for at least 6 months compared to untreated.

Selecting the optimal dosage for treatment of degeneration is also imperative for the clinical setting. Based on our results, no single dose of *NR2E3* was better across the board or caused any gross abnormalities, although the low dose had more robust rescue of ERG responses for all timepoints. Thus, the low dose of *NR2E3* therapy is the optimal choice for treatment in *Rho*^*P23H+/*^^−^ as it will slow degeneration for at least 6 months and likely prompt the lowest immune response.

Overall, the results of this study demonstrate the ability of various doses of AAV5-*hNR2E3* administered during early/intermediate progression to slow retinal degeneration in the *Rho*^*P23H+/*^^−^ mouse for a minimum of 6 months. Overexpression of *NR2E3* can restore homeostasis in the *Rho*^*P23H+/*^^−^ mouse by resetting key *Nr2e3*-associated pathways including the ER stress pathway therefore reducing degeneration and improving photoreceptor function and survival. The results of this study strongly support the potential of *NR2E3* as a gene modifier therapy capable of treating retinal degeneration in non-*NR2E3*-associated RP. Given that *NR2E3* likely slows degeneration, a booster may be necessary at minimum 6 months after initial treatment to maintain reduced degeneration. Future studies can evaluate the efficacy of a booster in a longitudinal study, and the efficacy of combination therapies. Further studies will examine the efficacy of *NR2E3* therapy in other non-*NR2E3*-associated forms of RP. This pharmacology study is translational to dosage selection, longitudinal effectiveness, and disease treatment stage for AAV5-*hNR2E3* therapy in clinical trials currently underway for various forms of RP and Leber Congenital Amaurosis (ClinicalTrials.gov Identifier: NCT05203939).

### Supplementary information

Supplementary Figure Legends

Supplemental Figure 1

Supplemental Figure 2

## Data Availability

All data used in this paper has been presented in the figures and supplementary files.
