# Supplementary material for: Preclinical dose response study shows NR2E3 can attenuate retinal degeneration in the retinitis pigmentosa mouse model RhoP23H+/−
Source: Gene Ther. 2024 Jan 26;31(5-6):255–62. doi: 10.1038/s41434-024-00440-6 (PMC11090815; doi:10.1038/s41434-024-00440-6)
Supplement: Supplementary file 1 — Supplementary Figure Legends [file 41434_2024_440_MOESM1_ESM.docx]

**Supplementary Figure Legends**

**Supplemental Figure 1. Treatment with AAV5-*hNR2E3* shows no adverse phenotypic changes in *Rho^P23H+/-^* mice for all doses.** A. 1-month *Rho^P23H+/-^* control and C57BL/6J (B6) wild type control fundus was used for reference at all three age ranges. B. *Rho^P23H+/-^* animals injected at 1 month of age and assessed 1-, 3-, and 6-months post-injection and age matched untreated animals (ages 2, 4, and 7 months). Low Dose 1 × 10^8^ vg/eye, Mid Dose 1 × 10^9^ vg/eye, High Dose 4 × 10^9^ vg/eye. N=5.

**Supplemental Figure 2. Subretinal injection has no observable effects on control untreated *Rho^P23H+/-^* retinas.** A. No major differences were observed in the fundus of *Rho^P23H+/-^* animals that received either no injection, a mock injection, or mock with saline buffer injection in the contralateral (left) control eye 1-, 3-, and 6-months after injection (ages 2, 4, and 7 months). B. H/E staining revealed no effects of mock or mock with saline buffer control injections on *Rho^P23H+/-^* retinas compared to those that received no injection. GCL: Ganglion Cell Layer; INL: Inner Nuclear Layer; ONL: Outer Nuclear Layer. Scale bar = 50 µm. N ≥ 8.
