# Supplementary material for: Preclinical dose response study shows NR2E3 can attenuate retinal degeneration in the retinitis pigmentosa mouse model RhoP23H+/−
Source: Gene Ther. 2024 Jan 26;31(5-6):255–62. doi: 10.1038/s41434-024-00440-6 (PMC11090815; doi:10.1038/s41434-024-00440-6)
Supplement: Supplementary file 2 — Supplemental Figure 1 [file 41434_2024_440_MOESM2_ESM.pptx]

## Slide 1
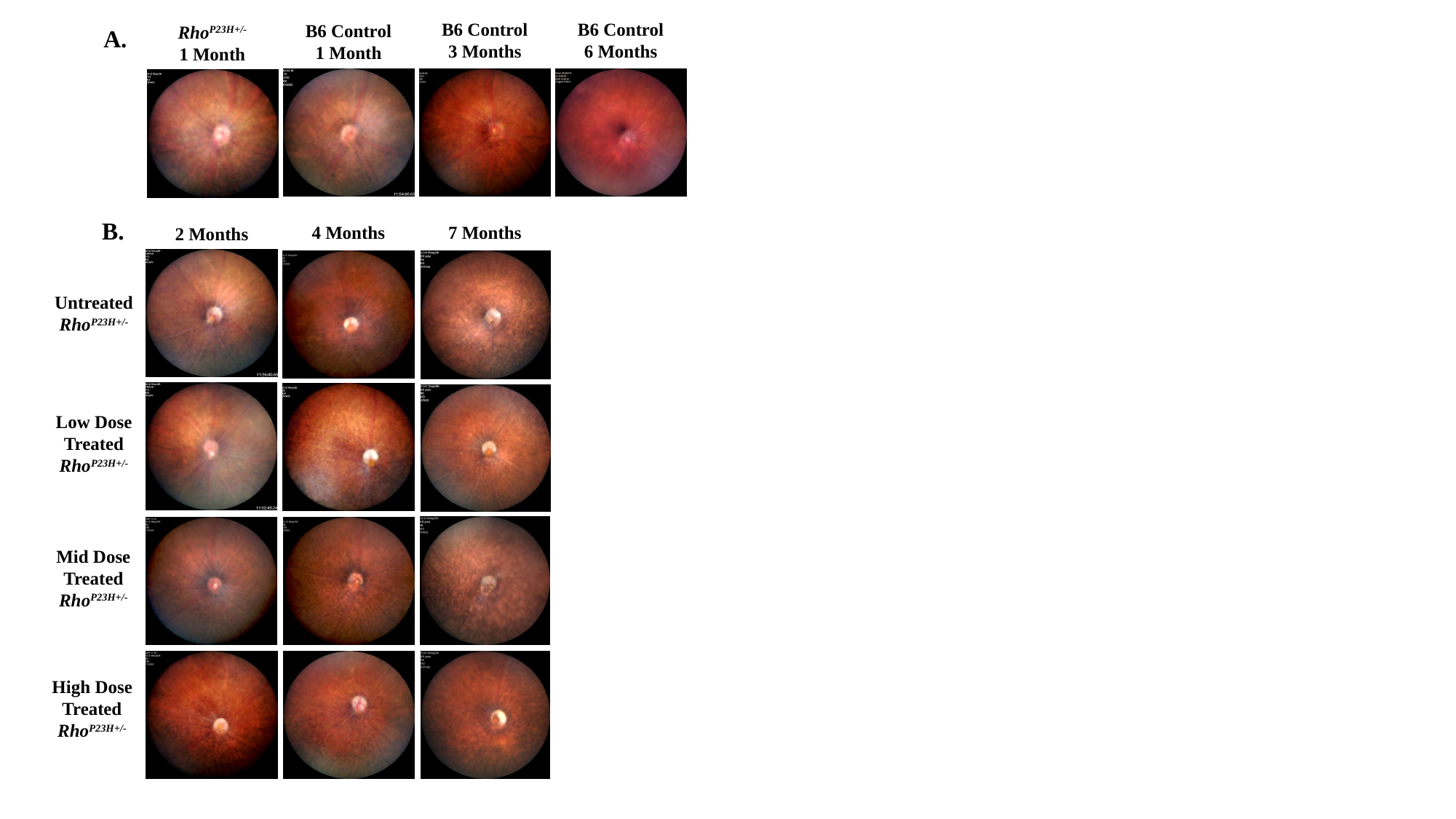

B6 Control
3 Months
B6 Control
6 Months
B6 Control
1 Month
RhoP23H+/- 1 Month
A.
B.
7 Months
4 Months
2 Months
Untreated
RhoP23H+/-
Low Dose Treated
RhoP23H+/-
Mid Dose Treated
RhoP23H+/-
High Dose Treated
RhoP23H+/-
