# Supplementary material for: Preclinical dose response study shows NR2E3 can attenuate retinal degeneration in the retinitis pigmentosa mouse model RhoP23H+/−
Source: Gene Ther. 2024 Jan 26;31(5-6):255–62. doi: 10.1038/s41434-024-00440-6 (PMC11090815; doi:10.1038/s41434-024-00440-6)
Supplement: Supplementary file 3 — Supplemental Figure 2 [file 41434_2024_440_MOESM3_ESM.pptx]

## Slide 1
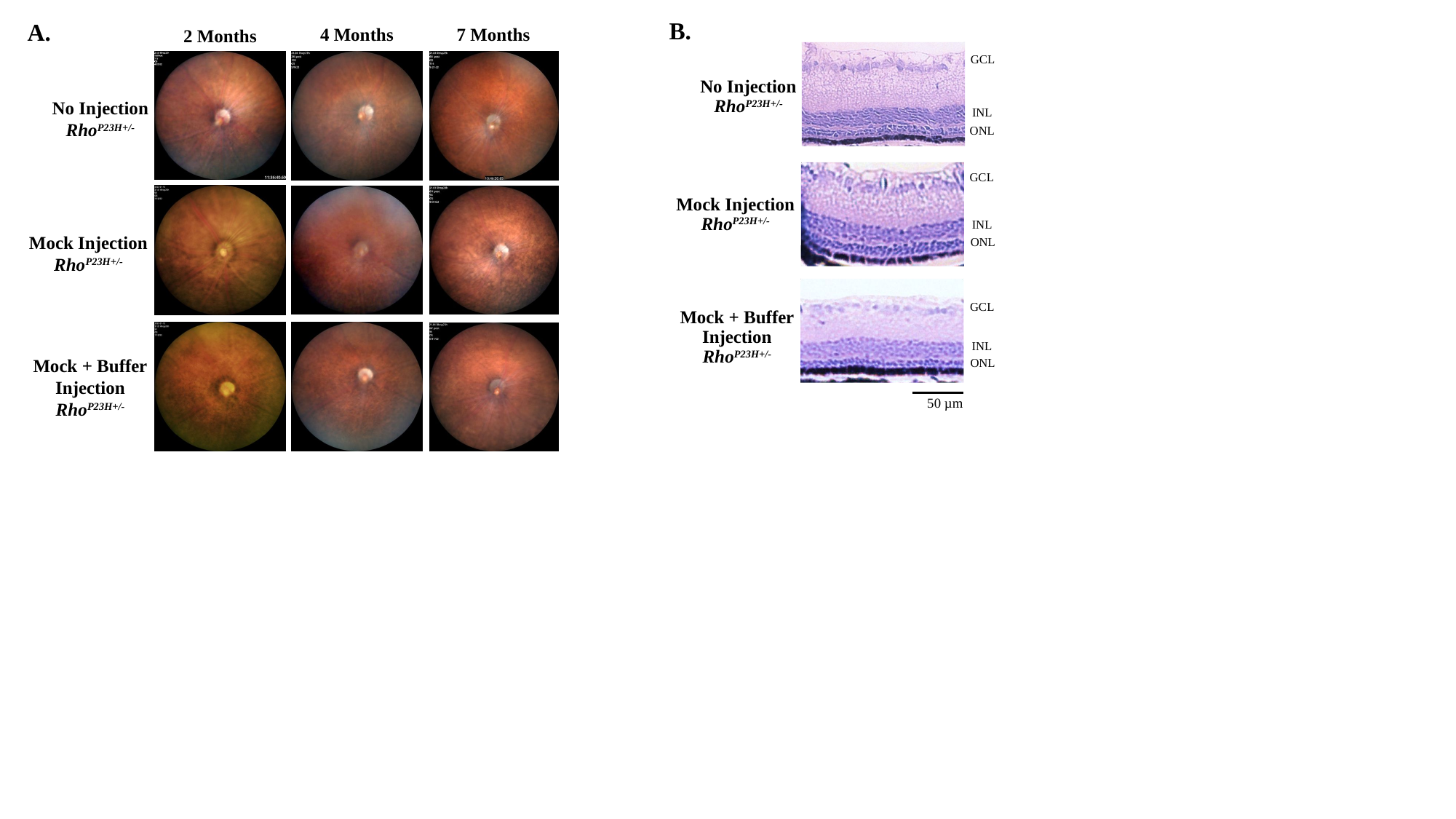

B.
GCL
No Injection RhoP23H+/-
INL
ONL
GCL
Mock Injection RhoP23H+/-
INL
ONL
GCL
Mock + Buffer Injection RhoP23H+/-
INL
ONL
50 µm
A.
7 Months
4 Months
2 Months
No Injection
RhoP23H+/-
Mock Injection
RhoP23H+/-
Mock + Buffer Injection
RhoP23H+/-
